# Migraine with aura in the locker room: three case reports

**DOI:** 10.1007/s10194-011-0369-6

**Published:** 2011-08-10

**Authors:** Ilenia Corbelli, Cataldo D’Amore, Stefano Caproni, Gabriela Cardaioli, Paolo Calabresi, Paola Sarchielli

**Affiliations:** Headache Centre, Neurologic Clinic, “Santa Maria della Misericordia” Hospital, University of Perugia, Sant’Andrea delle Fratte, 06158 San Sisto, Perugia Italy

**Keywords:** Migraine with aura, Physical activity, Football match, Exertion

## Abstract

It is well known that physical activity can aggravate the intensity of the headache, but the pathophysiological relationship between exertion and aura is still unknown. Anecdotal reports describe episodes of migraine preceded by head trauma and visual symptoms, migraine prodrome symptoms after unusually strenuous running with no subsequent head pain or recurrent attacks of hemiplegic migraine induced only by exertion. We describe the cases of three young men with recurrent episodes of migraine with aura occurring in the locker room shortly after a football match. Since the symptoms could mimic important pathologies in approximately 10% of these of headaches, it was mandatory to exclude a secondary form of headache in these patients. Several theories exist regarding the cause of primary exertional headache, but the pathogenesis of migraine triggered by physical activity has still not been identified. The present International Classification of Headache Disorders does not mention sport/exercise-induced migraine with aura episodes as primary headache. Since there are many cases described in the literature of migraine with aura triggered only by exercise, it may be helpful to specify, in the *typical aura with migraine headache* comments, that in some cases it can be exclusively triggered by sport/exercise.

## Introduction

Aura is defined as attacks of reversible focal neurological symptoms (visual, sensory and/or speech symptoms) that last more than 5 min and no longer than 1 h. Migraine-like headache usually occurs at the onset, during or follows aura symptoms [1]. Patients with motor weakness, according to the present classification, are classified separately as hemiplegic migraine [1].

It is well known that physical activity can lead to an aggravation of the intensity of the headache and a *primary exertional headache* is described in the current IHS classification under other primary headaches as a migraine-like headache unleashed by physical exertion [1].

We describe the cases of three patients with recurrent episodes of migraine with aura induced by physical activity. The relationship between exertion and aura is unknown and still debated; moreover, the International Classification of Headache does not, at present, include this subtype of headache [1].

## Cases presentation

Three young men (Case A 19 year-old, Case B 21 year-old, Case C 25 year-old) came to our observation for recurrent attacks of migraine-like headache of a pulsating quality and severe intensity, with nausea, photo- and phonophobia, that resolved spontaneously within 12 h if untreated. The attacks were preceded by reversible visual symptoms presenting as *fortification spectrum* and ipsilateral cheiro-oral paresthesia (Cases B and C only), that lasted for 15–40 min.

Onset of the symptoms occurred a few years earlier: 4 years for Cases A and C, 2 years for Case B.

In all cases the symptoms occurred when they were in the locker room: in Cases B and C shortly after a football match, but never after training; whilst in Case A the symptoms occurred occasionally after swimming pool and gym training or after participating in physical education activities at school.

No other subtypes of headache, or head trauma were reported by the patients.

The father of Case A and the mothers of Cases B and C suffered from episodic attacks of migraine without aura.

The patients’ medical history, physical and neurological examination did not suggest any other secondary disorder (Cases A and B are smokers). Case A also suffers from episodic migraine without aura according to the current International Classification of Headache Disorders (ICHD-II) Criteria [1].

The patients, however, underwent extensive diagnostic investigation: brain magnetic resonance with angiographic sequences, electroencephalography, vertebral and carotid echo-doppler, complete ocular examination, electrocardiography, transthoracic echocardiography and complete blood cell count, general chemistry profile, coagulation studies, protein C, protein S, antithrombin III, anticardiolipin antibody, erythrocyte sedimentation rate, C-reactive protein, antinuclear antibody and thyroid function test. The results of the laboratory tests and determinations were normal.

## Discussion

The prevalence of sport- and exercise-related headaches is estimated to be about 30–35% (15% of which are migrainous headaches) [2, 3]. In the study of Williams and Nukada [3], the reported subtypes of headache were: effort migraine, trauma-induced migraine, effort/exertion headache and post-traumatic headache; all effort and trauma-triggered migraine patients (11 and 8, respectively, of 129 patients) reported aura symptoms before the headache (visual and/or sensory disturbances). However, the precise epidemiology of this phenomenon is unknown [4].

According to ICHD-II criteria, the headache subtype presented by the patients fulfilled criteria for *typical aura with migraine headache* (ICHD-II code: 1.2.1) [1] and for *primary exertional headache* (pulsating headache, lasting from 5 min to 48 h, brought on by and occurring only during or after physical exertion; ICHD-II code: 4.3) [1].

Since these symptoms could mimic, in approximately 10% of these headaches, important pathologies, such as carotid dissection, artero-venous malformation, cerebral venus sinus thrombosis, seizures, subarachnoid or intraparenchymal haemorrhage [5], it was therefore mandatory to exclude a secondary form of headache in these patients.

There are anecdotal reports of episodes of migraine preceded by head trauma and visual symptoms (with a past history of non-sports-related migraine) [6], migraine prodrome symptoms after unusually strenuous running with no subsequent head pain [7] or recurrent attacks of hemiplegic migraine induced only by exertion [8].

To date, the pathogenesis of *primary exertional headache* is unidentified and it is still under debate; several theories regarding the cause have been proposed, such as, migraine triggered by physical effort particularly evident in aerobic exercise, altitude and hot weather, suggesting that low oxygen tension may trigger migraine by an as yet unknown mechanism [9]. Moreover, it has been postulated that a dilatation of pain-sensitive venous sinuses, at the base of the brain, as a result of increased cerebral arterial pressure, is induced by the exertion. In contrast to the hypothesized pathophysiology of the *cough headache* (ICHD-II code: 4.2), a similar vascular and hemodynamic pathogenesis is supposed to be shared by both *primary exertional headache and primary headache associated with sexual activity* (ICHD-II code: 4.4) [1], due to the similar age at onset, male predominance, pain characteristics and response to treatment [10].

The pathophysiological correlate of migraine aura is cortical spreading depression (CSD). In our patients, exertion seems to precipitate the aura. It has been suggested that the combination of exertion-induced hyperventilation and the subsequent hypocapnia with respiratory alkalosis and hypomagnesemia predisposes these patients to cerebral vasoconstriction during exertion lowering the threshold for the development of CSD or aura in migraineurs [8].

The present ICHD-II classification does not mention sport/exercise-induced migraine with aura episodes as primary headache, although there are many reports of attacks of migraine with aura shortly after practicing. Therefore, in case of patients suffering from this particular type of headache, there is currently the need to make a double diagnosis (*typical aura with migraine headache* and *primary exertional headache*; ICHD-II codes: 1.2.1 and 4.3), or to add a specifier (e.g. migraine with aura triggered by exercise). Since there are many cases described in the literature of migraine with aura triggered only by exercise, to faciliate the diagnosis it may be helpful to specify, in the *typical aura with migraine headache* comments, that in some cases it can be exclusively triggered by sport/exercise.
